# Tissue microarrays for testing basal biomarkers in familial breast cancer cases

**DOI:** 10.1590/S1516-31802007000400007

**Published:** 2007-07-01

**Authors:** Rozany Mucha Dufloth, Irina Matos, Fernando Schmitt, Luiz Carlos Zeferino

**Keywords:** Breast neoplasms, Biological markers, Genetic markers, Immunohistochemistry, Inborn genetic diseases, Câncer da mama, Marcadores biológicos, Marcadores genéticos, Imunohistoquímica, Doenças genéticas inatas

## Abstract

**CONTEXT AND OBJECTIVE:**

The proteins p63, p-cadherin and CK5 are consistently expressed by the basal and myoepithelial cells of the breast, although their expression in sporadic and familial breast cancer cases has yet to be fully defined. The aim here was to study the basal immunoprofile of a breast cancer case series using tissue microarray technology.

**DESIGN AND SETTING:**

This was a cross-sectional study at Universidade Estadual de Campinas, Brazil, and the Institute of Pathology and Molecular Immunology, Porto, Portugal.

**METHODS:**

Immunohistochemistry using the antibodies p63, CK5 and p-cadherin, and also estrogen receptor (ER) and Human Epidermal Receptor Growth Factor 2 (HER2), was performed on 168 samples from a breast cancer case series. The criteria for identifying women at high risk were based on those of the Breast Cancer Linkage Consortium.

**RESULTS:**

Familial tumors were more frequently positive for the p-cadherin (p = 0.0004), p63 (p < 0.0001) and CK5 (p < 0.0001) than was sporadic cancer. Moreover, familial tumors had coexpression of the basal biomarkers CK5+/p63+, grouped two by two (OR = 34.34), while absence of coexpression (OR = 0.13) was associated with the sporadic cancer phenotype.

**CONCLUSION:**

Familial breast cancer was found to be associated with basal biomarkers, using tissue microarray technology. Therefore, characterization of the familial breast cancer phenotype will improve the understanding of breast carcinogenesis.

## RESUMO

**CONTEXTO E OBJETIVO:**

As proteínas p63, p-cad e CK 5 são expressas em células basais/mioepiteliais da mama. Entretanto a expressão dessas proteínas no câncer esporádico e familiar ainda não é bem conhecida. O objetivo do estudo foi estudar essas proteínas no câncer de mama, utilizando a técnica de *tissue microarray*, assim como ER e HER2.

**TIPO DE ESTUDO E LOCAL:**

Estudo transversal, realizado no Centro de Atenção Integral à Saúde da Mulher, Universidade Estadual de Campinas, Brasil, e no Instituto de Patologia e Imunologia Molecular da Universidade do Porto, Portugal.

**MÉTODOS:**

O estudo analisou a expressão das proteínas p63, CK 5, p-cad, ER e HER2 numa série de 168 casos de câncer de mama. Os critérios utilizados para identificar as mulheres com alto risco foram os do Breast Cancer Linkage Consortium.

**RESULTADOS:**

A série de câncer familiar foi freqüentemente mais positiva para as proteínas basais p-cadherin (p = 0,0004), p63 (p < 0,0001) e CK 5 (p < 0,0001) que o câncer esporádico. A presença da co-expressão das proteínas basais CK 5+/p63+, agrupados dois a dois, foi associada com o fenótipo do câncer familiar (odds ratio, OR = 34,34), enquanto que sua ausência foi com o câncer esporádico (OR = 0,13).

**CONCLUSÕES:**

O câncer da mama familiar está associado aos marcadores de células basais proteínas p63, p-cad e CK 5, utilizando-se a técnica de *tissue microarray*. Por fim, parece legítima a interpretação destes resultados como mais uma evidência que suporta a hipótese da existência de células precursoras do câncer familiar da mama. O conhecimento dos perfis de expressão destas células, bem como das vias de sinalização envolvidas, beneficiarão o entendimento da carcinogênese mamária.

## INTRODUCTION

The understanding of breast biology and pathology is currently based on a concept of two cell types: glandular or luminal cells and myoepithelial cells.^1^ These cells have a common origin, arising from totipotent progenitor cells located in a suprabasal compartment between the myoepithelium and the luminal layer.^2^ Recent studies on breast cancer genetic expression using microarrays of complementary DNA (cDNA) have made it possible to distinguish two principal classes of tumor: one with the characteristics of basal cells and the other with the characteristics of luminal cells.^3-6^

The majority of sporadic breast cancer cases originate from luminal epithelial cells, and this finding is supported by morphological, biochemical and molecular evidence.^4,5,7,8^ A previous study carried out by our group showed that it is possible to characterize a basal breast cancer phenotype using the following markers: p63, cytokeratin 5 (CK5) and p-cadherin (p-cad).^9^ Essentially, cytokeratins 5 (CK5), 6 and 14 are recognized as basal forms of cytokeratin, whereas cytokeratins 8, 18 and 19 are expressed by glandular or luminal cells.^10,11^

The protein p63 is a homologous nuclear transcription factor of p53 and is necessary for breast development, as shown in experimental studies with knockout rats. The Tp63 gene encodes at least six different isoforms, and one of these (ΔNp63) is expressed in the basal cell population of the epithelium.^12^ Immunohistochemical studies have shown that p63 protein expression takes place in the nuclei of normal adult epithelial progenitor/basal cells, and that the predominant isoform is ΔN-p63α. Expression of this protein is lost with differentiation of the progenitor cells into luminal cells.^13,14^

p-cadherin is a glycoprotein that, in breast ducts and ductal-terminal units, is only expressed by myoepithelial and basal cells.^15,16^ Some studies have shown an association between p-cadherin expression in breast carcinomas and a myoepithelial embryonic and stem-cell-like phenotype.^17-19^

Thus, p63, p-cad and CK5 proteins are consistently expressed by the basal and myoepithelial cells of the breast,^10-13,20,21^ although the expression of these proteins in sporadic and familial breast cancer has yet to be fully defined. Immunohistochemical profiles in such cases have become easier to determine with the advent of tissue microarray technology.

## OBJECTIVE

The objective of this study was to evaluate the expression of basal biomarkers such as p63, p-cadherin and CK5, as well as estrogen receptor (ER) and Human Epidermal Receptor Growth Factor 2 (HER2), in a series of familial breast cancer cases, using tissue microarray technology (TMA).

## MATERIALS AND METHODS

### Informed consent

Clinical information, pathology reports, slides and paraffin blocks were obtained with the informed consent of patients, under the guidelines and approval of the Medical Research Ethics Committee of the School of Medical Sciences, Universidade Estadual de Campinas (Unicamp), and the National Commission for Research Ethics (Comissão Nacional de Ética em Pesquisa, Conep).

### Patient selection

168 women with breast carcinoma who were undergoing treatment at the Breast Cancer Outpatient Clinic of the Women's Comprehensive Healthcare Center (Centro de Atenção Integral à Saúde da Mulher, CAISM/Unicamp) were identified and invited to participate in this study. The criteria for identifying women at high risk were based on those of the Breast Cancer Linkage Consortium,^22,23^ as follows: early onset (less than 45 years old) and/or bilaterality; more than three cases of breast cancer and more than one case of ovarian cancer in the family; more than two first-degree relatives involved and cases of male breast cancer.

### Tissue microarray (TMA) construction

The TMA was constructed by acquiring 2.0 mm biopsy cores from representative areas of 168 tumors (TMA builder ab1802, Abcam®, Cambridge, United Kingdom).

**Figure 1A and 1B.**
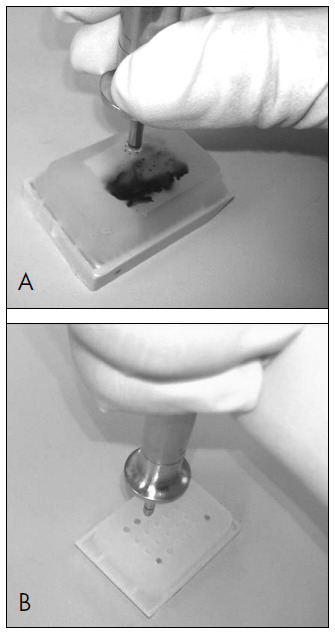
Construction of tissue microarray blocks, each containing 24 tissue biopsy cores.

Eleven TMA blocks (Figures 1A and 1B) were constructed, each containing 24 tissue cores arranged in a 4 × 6 sector. In each TMA block, non-neoplastic breast tissue cores were also included as controls. After construction, 2 μm tissue sections were cut and placed on Superfrost® Plus glass slides. One hematoxylin and eosin-stained 4 μm section from each block was reviewed to confirm the presence of morphologically representative areas of the original lesions.

### Immunohistochemistry

Immunohistochemical staining was performed using the streptavidin-biotin-peroxidase technique (Lab Vision Corporation, Fremont, California, United States) on each set of 11 glass slides comprising the TMAs. Antigen retrieval was performed by incubating the TMA sections in an antigen unmasking solution at pH 6.0 (Vector Laboratories, Inc., Burlingame, California, United States), or in ethylenediaminetetracetic acid (EDTA), pH 8 (Lab Vision) at 98º C. The antigen retrieval time, antibodies, dilutions and suppliers are listed in Table 1. After washing in phosphate buffer solution (PBS), endogenous peroxidase activity was blocked by incubating the slides in a 3% hydrogen peroxide solution in methanol (Merck, Germany). The slides were incubated with blocking serum (Lab Vision) for 10 minutes and then incubated with the specific antibody. Immunostaining was performed overnight at 4º C (p-cadherin and CK5) or for one hour at room temperature (HER2, ER, and p63). After washing, the slides were incubated with biotinylated secondary antibody, followed by streptavidin-conjugated peroxidase (Lab Vision). Diaminobenzidine was used as a chromogen. The tissues were then counterstained with hematoxylin, and cover slips were attached using a permanent mounting solution (Zymed, San Francisco, California, United States).

**Table 1.** Antibodies used in immunohistochemical study of breast cancer tumors

Biomarker	Antibody	Clone	Dilution	Origin
CK5	Mmab	XM26	1:80	Neomarkers, USA
p-cadherin	Mmab	56	1:50	BDTransduction, USA
p63	Mmab	4A4	1:150	Neomarkers, USA
Erα	Rmab	SP-1	1:20	Neomarkers, USA
HER2	MMab	NCL-L-CB11	1:60	Novocastra, UK

The immunoreactions were classified by estimating the percentage of tumor cells showing characteristic staining. In non-neoplastic breast tissues, p63 showed nuclear positivity in myoepithelial cells. p-cadherin presented distinctive membranous and occasionally cytoplasmic immunoreactivity in non-neoplastic myoepithelial cells. CK5 staining was present in the myoepithelial cells of breast lobules and ducts. Two pathologists (RD and FS) evaluated the immunohistochemical staining. Because non-neoplastic mammary secretory cells do not express p-cadherin, either membranous or cytoplasmic immunoreactivity was considered positive when more than 10% of the neoplastic cells expressed this marker.^12,24^ Similarly, we adopted the same cutoff value for nuclear p63 and ER reactivity.

To evaluate HER2, the percentage of cells with membranous staining and the intensity of the staining were assessed. HER2 was evaluated according to the four-category system (0-3+) and was considered positive when 3+ was attributed. We compared our HER2 results with fluorescence *in situ* hybridization (FISH) information that had previously been obtained. Out of the 73 tumor cases with FISH information available, 27 were simultaneously positive and 39 were simultaneously negative. Only seven tumors presented discordant information, of which five were HER2-positive and FISH-negative, and two were HER2-negative and FISH-positive. In cases of discordance, the FISH results were deemed to prevail.

### Statistical analysis

Data entry was carried out in the Excel software program (Microsoft) and then the data were exported to the StatView statistical analysis program, version 5.0 (SAS Institute Inc., Cary, North Carolina, United States). The relationships between the expression patterns of the molecular biomarkers were evaluated by constructing contingency tables and consequently applying the chi-squared test. The associations were considered statistically significant when p < 0.05.^25^

## RESULTS

### TMA validation

To validate the immunohistochemical analysis of the TMA, the ER expression for the sporadic cancers that was obtained in this study was compared with the data in the patients’ clinical records. Out of the 118 cases studied, 111 were concordant (86 were simultaneously positive and 25 were simultaneously negative). Only three cases presented discordant information and four could not be interpreted. The high percentage of concordance (97.4%) justified the subsequent analysis.

### Immunohistochemical profile of familial and sporadic breast cancer cases

**Figure 2.**
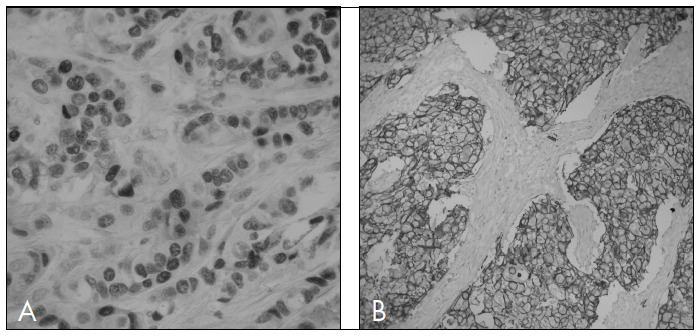
Sporadic breast carcinoma profile. A) ER positivity; B) HER2 positivity.

**Figure 3.**
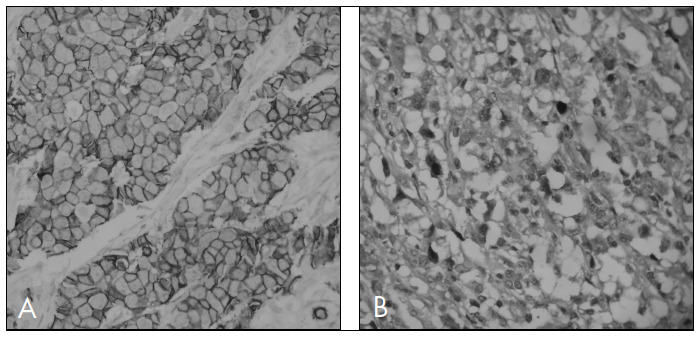
Familial breast carcinoma profile. A) p-cadherin positivity; B) p63 positivity.

Immunohistochemical analysis was conducted on each group of 11 slides that made up the TMAs for p63, p-cadherin, CK5, ER and HER2. The results are presented in Table 2 and Figures 2 and 3.

**Table 2.** Results from immunohistochemical staining on tissue microarray of breast cancer tumors

Biomarker	Interpretable biopsy coresn	Positive staining n (%)	Negative staining n (%)
p-cadherin	166	121 (73%)	45 (27%)
CK5	149	33 (21%)	116 (78%)
p63	154	31 (20%)	123 (78%)
ER	166	121 (73%)	45 (27%)
HER2	162	56 (34%)	106 (65%)

The familial breast cancer phenotype was more frequently positive for the basal biomarkers p-cadherin (p = 0.0004), p63 (p < 0.0001) and CK5 (p < 0.0001) than was the sporadic cancer phenotype. To evaluate the association between coexpression of basal biomarkers and the type of cancer, whether familial or sporadic breast cancer, the tumors were divided into six groups combining coexpression or lack of coexpression of the p63, p-cadherin and CK5 proteins. The presence of coexpression of the basal biomarkers CK5+/p63+ (odds ratio, OR = 34.34), grouped two by two, was associated with the familial breast cancer phenotype, while the absence of coexpression of the basal biomarkers CK5-/p63- and CK5-/p-cadherin- (OR = 0.13) was associated with the sporadic cancer phenotype. All the cases of basal phenotype (ER-/HER2-) were familial breast cancers (Table 3).

**Table 3.** Immunohistochemical profiles of familial (FH+) and sporadic (FH-) breast cancer phenotypes, as seen on tissue microarray

	FH (+) n (%)	FH (-) n (%)	OR (95% CI)	p (chi-squared test)
Only p63+	19 (40%)	12 (11%)	5.37 (2.16-13.53)	< 0.0001
Only CK5+	23 (49%)	10 (9.8)	8.42 (3.43-23.15)	< 0.0001
Only p-cad+	25 (51%)	21 (22%)	3.72 (1.67-8.36)	0.0004
p-cad+ and p63+	12 (25%)	3 (3%)	9.89 (2.39-47.23)	< 0.0001
p-cad- and p63-	15 (33%)	63 (69%)	0.22 (0.10-0.51)	< 0.0001
CK5+ and p63+	12 (26%)	1 (1%)	34.2 (4.33-731.14)	< 0.0001
CK5- and p63-	17 (36%)	80 (80%)	0.13 (0.06-0.31)	< 0.0001
p-cad+ and CK5+	14 (30%)	6 (7%)	6.46 (2.07-20.9)	0.0003
p-cad- and CK5-	14 (29%)	68 (76%)	0.13 (0.05-0.30)	< 0.0001
Only ER-	19 (38%)	26 (22%)	0.45 (0.21-0.99)	0.03
Only HER2 negative	37 (77%)	42 (42%)	0.22 (0.09-0.52)	< 0.0001
**Total number of cases (n)**	**50**	**118**		

FH (+): familial history positive; FH (-): familial history negative.

Positive cases were those that confirmed the profile under analysis. All others were considered negative.

## DISCUSSION

The objective of this study was to evaluate the expression of basal biomarkers such as p63, p-cadherin and CK5 in a series of familial breast cancer cases, using TMA. Familial breast cancer was characterized by the expression of these biomarkers. On the other hand, basal biomarker expression was significantly lower in cases of the sporadic cancer phenotype.

The studies with cDNA microarrays carried out by Perou et al.^3^ and de Sorlie et al.^6^ involved more than 8,000 genes from which different phenotypes of breast cancer were characterized. This scenario points towards the existence of strong gene interaction in the carcinogenic process, although this technology is not yet applicable in clinical practice. It is necessary, however, to identify markers that represent these genetic spectrums, using techniques that are currently available and are applicable in clinical practice.

This study therefore makes a contribution in the sense that it shows that the proteins associated with the basal cell phenotype are much more frequently present in familial breast cancer phenotype, and that they are much more frequently absent in the cancers that are considered sporadic and which follow the luminal pattern.

The estrogens that bind to the ER located in the cell nucleus, and the growth factors that bind to the HER2 protein located in the cell membrane represent two different routes for cell proliferation stimuli originating outside the cell. Therefore, it is possible to hypothesize that, when these lesions do not express ER or HER2 protein, the stimuli for cell proliferation would be determined by factors inside the cell or at least would be less dependent on external stimuli. Thus, the development of ductal carcinoma *in situ* (DCIS) would be more heavily dependent on external factors modulated by ER and HER2, while the progression to invasive cancer would be less dependent on this route, possibly because of the progressive accumulation of genetic alterations that occur in the malignant cells.^26-29^

Perou et al.^3^ characterized basal-subtype breast cancers as ER and HER2-negative. Therefore, these would be the cancers in which cell proliferation would depend less on external factors. Several studies have shown that women with the breast cancer 1 (BRCA1) gene mutation present breast cancer with a genetic expression pattern that is compatible with the basal subtype.^5,30-33^ However, the multistage process of carcinogenesis in the breast epithelium of women with genetic abnormalities who are highly susceptible to carcinogenesis would not depend on ER or HER2 protein expression, since the stimuli required for cell proliferation would be determined by the abnormalities present in the cell genome. Obviously, this explanation does not exclude the possibility that these cells may also have ER and HER2 protein expression.

Foulkes and colleagues^26,32^ studied this phenotype and found that around 40% of basal cancers presented a mutation in the BRCA1 gene, thus suggesting that this gene has a regulatory function in the progenitor cells of the breast and that it promotes the orderly transition of the cells to the glandular epithelial phenotype. Mutation of this gene would lead to interruption of the differentiation process, and the cells would remain stagnant in a primitive basal phenotype with no proliferation control.

The results from our study are in agreement with this theory on carcinogenesis, since they show that the familial breast cancer phenotype is more frequently ER (-) and HER2 protein (-) than are sporadic cancers. Moreover, the p63, p-cadherin and CK5 biomarkers showed that familial breast cancer cases more frequently present expressions compatible with the basal phenotype. As a rule, the sporadic cancer phenotype presents lower frequency of expression of these biomarkers. The remainder of our results are in full accordance with those in the literature.

## CONCLUSIONS

This familial cancer case series was found to be more frequently positive for the basal biomarkers p-cadherin, p63 and CK5, using tissue microarray technology. The scientific value of TMA was demonstrated through this study, even though the number of specimens was low. Many of the phenotypic features of familial cancer tumors might also be found in putative breast stem cells. Therefore, characterization of the familiar breast cancer phenotype will improve the understanding of breast carcinogenesis.
